# Sensitivity and specificity of the question “do you have any concerns regarding your mouth related to undergoing surgery?” for predicting perioperative oral health problems in patients with primary esophageal and lung cancer: a retrospective observational study

**DOI:** 10.1186/s13741-024-00394-8

**Published:** 2024-05-06

**Authors:** Aiko Yoshitomi, Yoshihiko Soga, Reiko Yamanaka-Kohno, Hiroshi Morimatsu

**Affiliations:** 1 Division of Hospital Dentistry, Okayama University Hospital, 2-5-1, Shikata-Cho, Kita-Ku, Okayama, 700-8558 Japan; 2 Perioperative Management Center, Okayama University Hospital, Okayama, Japan; 3 Department of Anesthesiology and Resuscitology, Okayama University Graduate School of Medicine, Dentistry, and Pharmaceutical Sciences, Okayama, Japan

**Keywords:** Sensitivity, Specificity, Perioperative, Oral management, Screening, Question

## Abstract

**Background:**

Perioperative oral management contributes to the prevention of dental/systemic complications. However, a professional dental checkup before surgery is generally not performed and relies on the patient’s answer to a simple question by medical professionals other than dentists: “Do you have any concerns regarding your mouth related to undergoing surgery?” Here, we evaluated the sensitivity and specificity of this question for predicting perioperative oral health problems in patients with primary esophageal and primary lung cancer.

**Methods:**

We performed an oral cavity check in all patients before scheduled surgery for primary esophageal and lung cancer. A total of 183 patients were enrolled (M, 112; F, 71; 24–88 years, median, 69 years), consisting of 61 with primary esophageal cancer (M, 46; F, 15; 24–85 years, median, 69 years) and 122 with primary lung cancer (M, 66; F; 56; 33–88 years, median, 69 years). All subjects provided a response to this question, and an oral cavity check was performed by dentists. The sensitivity and specificity of this question for detecting oral health problems were evaluated retrospectively.

**Results:**

Overall sensitivity and specificity for detecting oral health problems were 0.263 and 0.898, respectively. There were no significant differences by sex or disease (primary esophageal or lung cancer).

**Conclusion:**

This simple question has low sensitivity but high specificity for detecting oral health problems. Although challenging to detect surgical patients with oral health problems by simply asking questions, the results indicated that patients with oral complaints are more likely to have problems during surgery.

**Supplementary Information:**

The online version contains supplementary material available at 10.1186/s13741-024-00394-8.

## Introduction

Perioperative oral management can contribute to the prevention of dental/systemic complications. Dental injury is a well-known complication of airway management in patients under general anesthesia. A recent review by Sahni (2016) showed reported incidences of dental trauma in patients undergoing general anesthesia of 1 in 2073 (0.05%) and 1 in 2805 (0.04%) (Newland et al. 2007; Warner et al. 1999). In a retrospective study, Rosenberg (1989) reported an incidence of 1:1000 endotracheal intubations. Prospective studies, however, such as those conducted by Chen et al. (1990), reported a higher rate of occurrence in the range of 12.1%. Many dental injuries can occur, including enamel fracture, subluxation, luxation, avulsion, crown fracture, crown and root fracture, and damage to dental restorations and prostheses. Gaudio et al. (2010) reported that the avulsion of permanent teeth in patients with severely mobile teeth accounted for 50% of total cases of iatrogenic dental trauma. Appropriate dental pre-examination and measures can contribute to patient safety.

From the perspective of infection control, oral care by dental staff has recently attracted attention for the prevention of in-hospital pneumonia. Ishimaru et al. (2018) analyzed Japan’s nationwide administrative claim database and identified a total of 509,179 patients who underwent resection of head and neck, esophageal, gastric, colorectal, lung, or liver cancer, and showed that oral care by a dentist was significantly associated with a decrease in risk of postoperative pneumonia.

From a nutritional perspective, the enhanced recovery after surgery (ERAS®) protocol, originating in the ERAS group organized by the European Society for Clinical Nutrition and Metabolism (ESPEN) in 2001, is representative of multidisciplinary strategies for accelerated postoperative recovery. In the ERAS® protocol (Fearon et al. 2005), oral nutrition intake during the perioperative period is among the main factors associated with accelerated postoperative recovery, avoiding starvation conditions as much as possible throughout the perioperative period. Pre- and early post-oral nutrition intake is practiced even after surgery. Therefore, dental staff could contribute due to their specialty, although they are not included in the original multidisciplinary approach.

Along with the worldwide revolution in perioperative management, Okayama University Hospital established a perioperative management center (PERiO) in 2008 to maintain safe, authentic, and high-quality perioperative management (Yasuhara et al. 2016; Soga et al. 2019). PERiO is comprised of anesthesiologists, surgeons, dentists/dental hygienists/dental technicians, nurses, physical therapists, pharmacists, dieticians, and medical engineers (Yasuhara et al. 2016; Soga et al. 2019). Dental staff participate extensively in the multidisciplinary approach to perioperative management (Yasuhara et al. 2016; Soga et al. 2019). Dentists, dental hygienists, and dental technicians perform perioperative management aimed at preventing acute odontogenic pain caused by pulpitis and/or acute odontogenic infection during the perioperative period, enabling patients to achieve oral nutrition/ingestion by dental treatment, preventing dental injury during orotracheal intubation, preventing postoperative pneumonia by maintaining oral hygiene, and enabling safe oral nutrition by appropriate eating/swallowing functional evaluation and rehabilitation (Soga et al. 2019).

At the time of writing, patients in the departments of gastrointestinal surgery, thoracic surgery, breast and endocrine surgery, and hepato-biliary-pancreatic surgery in our hospital are managed by PERiO. Some surgery departments in our hospital have reported the benefits of PERiO. Shimoda et al. (2016) investigated the impact of PERiO on clinical outcomes of 127 elderly patients undergoing thoracic surgery for resection of non-small cell lung cancer. Radical operations were performed significantly more frequently after than before the introduction of PERiO, while the postoperative complication rates remained similar (Shimoda et al. 2016). The duration of postoperative hospitalization was reduced, and the hospital income was increased after the introduction of PERiO (Shimoda et al. 2016). Yasuhara et al. (2016) investigated neurosurgical patients and noted that the PERiO system decreased the period from admission to surgery. As perioperative management with PERiO constitutes a multidisciplinary care bundle approach, it is difficult to determine the contribution of dental staff alone. However, Yamanaka et al. (2013) reported an esophageal cancer surgery patient without occlusal support who gained body weight after denture treatment. In our previous study, a dental checkup before surgery revealed that the condition of the oral cavity was poor in patients with esophageal cancer (Yamanaka-Kohno et al. 2022).

There are many benefits to the extensive participation of dental staff in perioperative management. However, in Japan, about 90% of dentists work in dental clinics and engage in community dental health care, with only about 3% of dentists working in hospitals, excluding educational institutes (i.e., dental schools of universities and dental colleges) (Japanese Ministry of Health 2014). Generally, most of the work of hospital dentistry is as secondary and tertiary medical institutions, and the number of dentists involved in improving the quality of hospital medical care is insufficient.

A routine professional dental checkup before surgery is generally not performed, and management relies on the patient’s answer to the simple question by medical professionals involved in the perioperative period other than dentists: “Do you have any concerns regarding your mouth related to undergoing surgery?” Although potentially important, the efficacy of this question for detecting oral health problems is not yet clear. However, a response indicating concern regarding potential oral issues will trigger a referral to a local dental care provider.

Currently, professional dental checkups before surgery at PERiO are not performed in all patients due to human resource issues, but all patients scheduled for surgery for primary esophageal and lung cancer are checked before surgery. In addition, the questionnaire before the dental visit elicits a yes/no response to the question, “Do you have any concerns regarding your mouth related to undergoing surgery?” This study was performed to evaluate the sensitivity and specificity of this simple question for predicting perioperative oral health problems in patients with primary esophageal and lung cancer.

## Subjects and methods

### Subjects

Patients with primary esophageal (including esophagogastric junctional) and lung cancer presenting to our perioperative management center between April 1, 2018, and October 15, 2018, were enrolled and examined retrospectively. In principle, we perform a professional dental checkup before surgery by dentists in all patients undergoing scheduled surgery for primary esophageal and lung cancer. A total of 183 patients was enrolled (M, 112; F, 71; 24–88 years, median, 69 years), consisting of 61 with primary esophageal cancer (including esophagogastric junctional cancer) (M, 46; F, 15; 24–85 years, median, 69 years) and 122 with primary lung cancer (M, 66; F; 56; 33–88 years, median, 69 years) (Table 1).

**Table 1:** Numbers of subjects and age groups by disease

Disease	Number			Age (years) (range, median)
	M	F	Total	
Primary esophageal cancer (including esophagogastric junctional cancer)	46	15	61	24–85, 69
Primary lung cancer	66	56	122	33–88, 69
Total	112	71	183	24–85, 69

### Questionnaire and timing

In our perioperative management center, all patients are interviewed by nurses for physical evaluation, explanation/guidance, and decision support for surgery and introduced to our multidisciplinary perioperative management. The roles of dental staff, as described in the introduction, are also explained. After the interview and explanation, patients come to the dentists of PERiO and are asked to provide a yes/no answer to the question, “Do you have any concerns regarding your mouth related to undergoing surgery?”.

### Criteria of problems

After obtaining the response to the question, dentists of PERiO perform dental checks, including X-ray examinations, for all referred patients to detect problems according to the following criteria.

- 1) *Preventing acute odontogenic pain caused by pulpitis and/or acute odontogenic infection during the perioperative period*
  Dental caries, which can cause odontogenic pain by acute pulpitis, and active odontogenic infection (mainly marginal/periapical periodontitis and pericoronitis) with abscess and/or pus discharge, possibly requiring administration of antibiotics, during the perioperative period (from admission to discharge) were regarded as problems.
- 2) *Decreasing the risk of postoperative pneumonia due to poor oral hygiene*
  The presence of food residue, calculus, and plaque in many areas and strong halitosis were regarded as problems.
- 3) *Enabling patients to achieve oral nutrition by dental treatment*
  Insufficient occlusion of the molars caused by missing teeth, occlusal pain due to marginal and/or periapical periodontitis, and ill-fitting dentures raising concerns about postoperative oral nutrition were regarded as problems.
- 4) *Preventing dental injury during orotracheal intubation*
  The dentist assumed an intubation operation and focused on the anterior maxillary teeth. Patients thought to require some form of treatment to prevent dental injury (e.g., tooth luxation because of severe periodontitis, damage to or detachment of dental restoration) during orotracheal intubation were regarded as having problems.

### Evaluation of the sensitivity and specificity of the question, “Do you have any concerns regarding your mouth related to undergoing surgery?”

The yes/no responses of patients to the question were complied, and the sensitivity, specificity, and reproducibility (Cohen’s Kappa coefficient) of the question for detecting oral health problems were calculated. Overall sensitivity, specificity, and reproducibility (Cohen’s Kappa coefficient) were calculated by sex, age group, and disease. Differences between sexes and between disease groups were examined with the chi-square test. Differences in the distribution of responses by age group were also analyzed with the chi-square test. Statistical analysis was performed using Stata SE 13.0 (StataCorp, College Station, TX, USA). In all analyses, *P* < 0.05 was taken to indicate statistical significance.

## Results

### Overall sensitivity and specificity of the simple question, “Do you have any concerns regarding your mouth related to undergoing surgery?”

All yes/no responses obtained from patients along with the decisions of dentists are shown in Table 2. The overall sensitivity and specificity of the question for detecting oral health problems were 0.263 and 0.898, respectively. Cohen’s Kappa coefficient was 0.157.

**Table 2:** Responses of patients to the question “do you have any concerns regarding your mouth related to undergoing surgery?” and the decisions of dentists

		Dentist decision		Total
		Intervention is desirable	No problem	
Response	Yes	25 (13.7%)	9 (4.9%)	34 (18.6%)
	No	70 (38.3%)	79 (43.2%)	149 (81.4%)
Total		95 (51.9%)	88 (48.1%)	183 (100%)

### Sensitivity and specificity by sex of the simple question, “Do you have any concerns regarding your mouth related to undergoing surgery?”

The yes/no responses of patients to the question and the decisions of dentists are shown according to sex in Table 3 (A and B). The results showed sensitivity and specificity of 0.212 and 0.950, respectively, in male patients and 0.326 and 0.786, respectively, in female patients. There were no significant differences in sensitivity or specificity according to sex (*P* = 0.209, chi-square test). Cohen’s Kappa coefficients were 0.170 in male patients and 0.097 in female patients, respectively.

**Table 3:** Responses of patients to the question “do you have any concerns regarding your mouth related to undergoing surgery?” and the decisions of dentists by sex

		Dentist decision		Total
		Intervention is desirable	No problem	
A. Male				
Response	Yes	11 (9.8%)	3 (2.7%)	14 (12.5%)
	No	41 (36.6%)	57 (50.9%)	98 (87.5%)
Total		52 (46.4%)	60 (53.6%)	112 (100%)
B. Female				
Response	Yes	14 (19.7%)	6 (8.5%)	20 (28.2%)
	No	29 (40.8%)	22 (31.0%)	51 (71.8%)
Total		43 (60.6%)	28 (39.4%)	71 (100%)

### Sensitivity and specificity by disease of the simple question, “Do you have any concerns regarding your mouth related to undergoing surgery?”

The yes/no responses of patients to the question and the decisions of dentists are shown according to disease in Table 4 (A and B). The results showed sensitivity and specificity of 0.316 and 1.000, respectively, in primary esophageal cancer and 0.228 and 0.862, respectively, in primary lung cancer (Table 5). Cohen’s Kappa coefficients were 0.258 in primary esophageal cancer and 0.093 in primary lung cancer, respectively (Table 5). There were no significant differences in sensitivity or specificity according to disease (*P* = 0.342, chi-square test).

**Table 4:** Responses of patients to the question “do you have any concerns regarding your mouth related to undergoing surgery?” and the decisions of dentists by the disease

		Dentist decision		Total
		Intervention is desirable	No problem	
A. Primary esophageal cancer				
Response	Yes	12 (19.7%)	0 (0.0%)	12 (19.7%)
	No	26 (42.6%)	23 (37.7%)	49 (80.3%)
Total		38 (62.3%)	23 (37.7%)	61 (100%)
B. Primary lung cancer				
Response	Yes	13 (10.7%)	9 (7.4%)	22 (18.0%)
	No	44 (36.1%)	56 (45.9%)	100 (82.0%)
Total		57 (46.7%)	65 (53.3%)	122 (100%)

**Table 5:** Sensitivity, specificity, and reproducibility of the question “do you have any concerns regarding your mouth related to undergoing surgery?” by disease

	Sensitivity	Specificity	Reproducibility (Kappa coefficient)
Primary esophageal cancer	0.316	1.000	0.258
Primary lung cancer	0.228	0.862	0.093

## Discussion

Overall, the simple question, “Do you have any concerns regarding your mouth related to undergoing surgery?” had low sensitivity and high specificity for detecting oral health problems in patients undergoing scheduled surgery for primary esophageal and lung cancer. By sex, the sensitivity was higher in female than male patients, although the values were both low (M: 0.212; F: 0.326), while specificity was relatively high in both sexes (M: 0.950; F: 0.786). There were no significant differences in sensitivity or specificity between patients with primary esophageal cancer and esophageal cancer. The reproducibility (Cohen’s Kappa coefficient) of the question for detecting oral health problems was poor.

As professional dental checkups before surgery oral cavity checks at PERiO are not performed in all patients due to human resource issues, all patients scheduled to undergo surgery for primary esophageal and lung cancer are checked before surgery. This study was conducted on these patients. Although detailed data are not shown, a total of 361 patients were referred for professional dental checkups before surgery during the study period, other than in cases of esophageal and lung cancer surgery, which were referred based on patient complaints, interviews, and detection of problems during preoperative consultation with the anesthesiologist. Although there was likely bias because not all interventions were available, 361 patients were seen for professional dental checkups before surgery during the study period. The overall sensitivity and specificity of the question for detecting oral health problems were 0.306 and 0.808, respectively. The trend was consistent with the results of this study, which included all patients scheduled to undergo surgery for primary esophageal and lung cancer.

In terms of sensitivity and reproducibility of the question for detecting oral health problems, safe and high-quality perioperative care requires not only questions but also expert evaluation of the oral cavity, and it seems necessary to assign dentists to hospitals to improve the quality of perioperative care. It should be noted that in the preoperative outpatient process in PERiO, the nurse explains the actual flow of the surgery, tracheal intubation, etc., and questions about the oral cavity are asked with a reasonable understanding of this information. Without this process, the sensitivity of the question for detecting oral health problems would likely be even lower.

On the other hand, this question had high specificity for detecting oral health problems, and medical staff should pay attention to this information. Patients who answer “yes” to this question in a hospital without a dentist should be consulted by an outside dental institution to resolve the problem if permitted by their general condition and surgical schedule. The universal health insurance system in Japan covers oral management at community dental institutions before surgery (Sekiya et al. 2021).

One limitation of this study was that the primary cancers in all surgical patients included in PERiO’s oral management were esophageal and lung cancers, so only these patient groups were included. Smoking is a risk factor for esophageal and lung cancer, consistent with the risk factors for periodontal disease. Based on a systematic review and meta-analysis, Wang et al. reported that smoking cessation decreases the risk of esophageal squamous cell carcinoma in a time-dependent manner (Wang et al. 2017). In another systematic review and meta-analysis, Lee et al. showed that the association of lung cancer with smoking is strong (Lee et al. 2012). Although the risk is lower in Japan than in Western populations (Wang et al. 2017; Lee et al. 2018), smoking is definitely also a risk factor for lung cancer in Japan. Smoking is also an important risk factor for the occurrence and progression of periodontal disease (Kinane and Chestnutt 2000). The sensitivity of the question for detecting oral health problems may have been increased because the study was conducted in a group of patients who were more likely to have poor oral health. However, it was worthwhile to determine the validity of such a simple question for a group of diseases with the same risk factors as cancer and poor oral health, with a high need for oral management before surgery. It may be helpful to examine and compare the sensitivity and specificity of this question in patients with other cancers in future studies.

In addition, there were limitations related to the retrospective nature of this study. However, a prospective manner may have been subject to bias due to awareness of the purpose of the study when asking the question. Therefore, a retrospective analysis of the responses to this question would ensure a reasonable degree of reliability.

In conclusion, the simple question, “Do you have any concerns regarding your mouth related to undergoing surgery?” had low sensitivity but high specificity for predicting perioperative oral health problems in patients with cancer. Although it is challenging to detect patients undergoing surgery who have problems with their oral health by asking questions alone, the results indicated that patients with oral complaints are more likely to have problems during surgery.

### Supplementary Information

**Additional file 1.** Raw data—primary esophageal cancer and raw data—primary lung cancer.

## Data Availability

The data supporting the conclusions of this article are provided in Additional file 1.
